# Everybody's Page

**Published:** 1888-03-03

**Authors:** 


### Everybody's Page.

The reason why distant objects appear closer just before rain is that the air gets damp then, and damp air is more transparent than dry.

There are seventeen quinine factories in the world—six in Germany, four in America, three in France, two in Italy, and two in England. The annual production amounts to about 4,250,000 ounces.

Dr. Weyl, of Berlin, who has been examining the various yellow colouring matters used for maccaroni, butter, liqueurs, etc., reports that dinitrocresol, known as saffron yellow, is highly poisonous; four grains having proved fatal to rabbits and guinea-pigs in a few minutes.

Dr. Blache states in the *Bulletin Thérapeutique* that in chronic and simple bronchitis, petroleum in teaspoonful doses before meals produces satisfactory results. In phthisis the experiment has not been made long enough to ascertain whether the results are permanently beneficial; but the petroleum is said to diminish the expectoration and to lessen its purulent character.

Our men seem to be hardier in these "degenerate days" than they were a century ago. Formerly gentlemen as well as ladies regarded the muff as an integral part of their winter attire; and no beau could enjoy that satisfactory consciousness of being well dressed, which the American damsel declared even religion could not compensate for losing, unless a small muff dangled from his neck by a silken ribbon.

The full complement of the adult or permanent set of teeth in man amounts to thirty-two in number—sixteen in the upper and sixteen in the lower jaw. Anatomists have generally considered forty-two instead of thirty-two to be the typical or normal number of teeth to be found in animals belonging to the mammalian class, among which man is included. They therefore hold that certain teeth in man and many other animals are suppressed, and thus never make their appearance. A knowledge of this diminution in the number of teeth in man is interesting, as it explains to some extent the occurrence of what is sometimes set down as a third dentition, as well as the occasional appearance of extra or supernumerary teeth in the mouth—such additional members in both cases merely indicating a tendency to return to the typical number.

An attempt is being made to form some of the hard-worked sempstresses of the East-end into a co-operative society. There were said to be no fewer than twenty-five thousand women in London who are slaving away their lives for a penny an hour, and are glad under existing conditions to get work at that rate of payment. The members of the Labour Aid Society have determined to do something towards ending this state of things. The promoters of the company, which proposes to ameliorate the sempstresses' condition, are nearly all business men, and their intention is to raise a small capital among benevolent people, and with this to start an association of needlewomen, who will be given work at fair trade prices and not at the starvation rates which the middlemen too often force the women to accept. The subscribers to the capital fund are to receive no profit on their investment, and all profits are to be carried to a new capital fund, which will, it is hoped, soon reach such a sum as would enable the company to return their subscriptions to the original shareholders. The shares will then be divided among the women forming the association, which will from that time be carried on upon a co-operative basis. Should the present attempt prove successful, an attempt will be made to found similar societies in all districts of the East-end.

It is said that bisulphide of carbon is successful in relieving neuralgia. From fifteen to twenty drops should be applied on cotton wool to the painful part, and covered with a piece of dry cotton. The application will cause a little pain, but this is temporary, and to get rid of the maddening pangs of neuralgia is worth more than a trifling sting.

Fish may be broadly divided into two classes—those which have fat distributed throughout the body, and those which have the fat gathered into one part—the liver. In the first class are comprised salmon, herring, eels, and mackerel, and these are undoubtedly more nutritious. The remaining white fish are included in the second class, and are, as a whole, the more easily digested.

There is no greater digestive error than engaging in brain work immediately after a heavy meal. It is as injurious as to do heavy manual work; both courses tending to draw the blood away from the stomach, where a larger than ordinary supply is required to assist digestion. When brain or physical work has to be done during the day, a light and easily-digested meal alone should be taken, and the chief meal left till after the work is done. But if the brain or body is chiefly to be taxed late in the day or at night, the chief meal should be taken in the middle of the day.

A little knowledge of chemistry has often, when possessed by a person of an experimental turn of mind, led to disastrous results; but total ignorance may be more dangerous still. Some time ago a nurse in a London hospital was cleaning a bottle which had contained glycerine. To facilitate matters she poured in some nitric acid, thereby unintentionally forming the explosive compound nitroglycerine. The bottle burst in her hands, and one piece flew up and struck her face with such violence that her cheek was badly cut and one eye seriously injured.

Socin speaks highly of oxide of zinc as an antiseptic. For the irrigation of deep wounds he recommends a one per cent. mixture with water; superficial open wounds should be washed with a ten per cent. mixture. Large raw surfaces, burns, contusions, etc., may be dusted with the oxide in the form of powder. He also advises, as a permanent dressing, a paste composed of fifty parts each of water and oxide of zinc, with five parts of chloride of zinc. When this dries it forms a thin coating over the wound, beneath which healing takes place, he finds, with unusual rapidity. It must be remembered, however, that oxide of zinc is almost useless when applied to a wound that has already become septic.

A bactericide is a bacterium killer, and if the latest researches of science point to correct conclusions, it is a substance that everybody ought to know a good deal about, and to use very largely. The enemy of the modern man is not war, nor pestilence, nor port wine, nor turtle soup, but the bacterium—a fellow of infinite variety. One kind of bacterium produces typhoid, another scarlet fever, another whooping-cough, another diphtheria, and so on. If only we could kill them, every one, what a happy world we should have! Kingzett's bactericide is theoretically perfect as a bacterium destructor. It consists of corrosive sublimate, a highly-energetic destroyer of low organisms, and peroxide of hydrogen, a powerful oxidising or burning agent. When bacteria, or the products, such as diphtheritic membranes, which are associated with them, are acted upon by either of these agents, they are rendered harmless; but when they are acted upon by both of them in combination, it may be assumed with certainty that they are absolutely destroyed. We have made a prolonged trial of Kingzett's bactericide, and are convinced that it is as practically efficacious as it is in compo sition theoretically perfect.

